# Occupational exposure to respirable crystalline silica and lung cancer: a systematic review of cut-off points

**DOI:** 10.1186/s12940-023-01036-0

**Published:** 2023-11-30

**Authors:** Julia Rey-Brandariz, Cristina Martínez, Cristina Candal-Pedreira, Mónica Pérez-Ríos, Leonor Varela-Lema, Alberto Ruano-Ravina

**Affiliations:** 1 Department of Preventive Medicine and Public Health, Universidade de Santiago de Compostela, C/ San Francisco s/n, Santiago de Compostela, 15782 Spain; 2 Consortium for Biomedical Research in Epidemiology and Public Health (CIBER en Epidemiología y Salud Pública- CIBERESP), Madrid, Spain; 3 Asturias Clinical Hospital, Oviedo, Spain; 4 Principality of Asturias Health Research Institute (Instituto de Investigación Sanitaria del Principado de Asturias-ISPA), Oviedo, Spain; 5 Health Research Institute of Santiago de Compostela (Instituto de Investigación Sanitaria de Santiago de Compostela-IDIS), Santiago de Compostela, Spain

**Keywords:** Respirable crystalline silica, Lung cancer, Occupational exposure, International agencies, Systematic review

## Abstract

**Background:**

Respirable crystalline silica (RCS) is associated with the development of lung cancer. However, there is uncertainty around the exposure threshold at which exposure to RCS may pose a clear risk for the development of lung cancer. The objective of this study was to review the cut-off points at which the risk of mortality or incidence of lung cancer due to occupational exposure to RCS becomes evident through a systematic review.

**Methods:**

We conducted a search in PubMed, including cohort and case-control studies which assessed various categories of RCS exposure. A search was also conducted on the webpages of institutional organizations. A qualitative data synthesis was performed.

**Results:**

Twenty studies were included. Studies that assessed lung cancer mortality and incidence displayed wide variability both in RCS exposure categories and related risks. Although most studies found no significant association for RCS exposure categories, it appears to be a low risk of lung cancer for mean concentrations of less than 0.07mg/m^3^. Regulatory agencies set annual RCS exposure limits ranging from 0.025mg/m^3^ through 0.1mg/m^3^.

**Conclusions:**

There is a wide degree of heterogeneity in RCS exposure categories, with most studies observing no significant risk of lung cancer for the lowest exposure categories. Cut-off points differ between agencies but are nonetheless very similar and do not exceed 0.1mg/m^3^.

**Supplementary Information:**

The online version contains supplementary material available at 10.1186/s12940-023-01036-0.

## Introduction

Crystalline silica is a mineral that occurs naturally in the earth’s crust and can assume a number of forms, with α-quartz being the most abundant [1]. Most exposure to this mineral takes place in a work environment. Occupations related with mining, iron foundries, construction, cement, glass, ceramic, quartz conglomerate, and all those involving earthmoving are jobs in which workers may be exposed to respirable crystalline silica (RCS) [1, 2]. It is estimated that several million workers are exposed to RCS in Europe [3] and that around 2 million construction workers could be exposed in the USA [4].

In 1997, the International Agency for Research on Cancer (IARC) classified RCS found in the work environment in the form of quartz or cristobalite as a Group 1 human carcinogen [5], and confirmed this classification in its subsequent monograph published in 2012 [1]. Despite there being epidemiologic evidence of a relationship between RCS exposure and lung cancer, there are aspects related with the carcinogenicity of RCS which are not clear and hinder the establishment of protective measures, even at a legislative level. One of the existing uncertainties surrounds the cut-off point that should be set to reduce the risk of lung cancer. In relation with this aspect, stress should also be laid on the difficulty of measuring exposure, because, while RCS can be measured in a specific workplace, workers do not always remain in the same place throughout the workday. Hence, attribution of exposure to a given worker according to the concentration found in a given place may under- or overestimate that particular worker’s real exposure. An additional complication is the frequent concurrence of lung cancer risk factors, such as smoking, which render it even more complicated to distinguish the specific risk associated with a concrete exposure to RCS.

While a number of systematic reviews have previously been conducted on RCS exposure and risk of lung cancer [6, 7], none has sought to assess from which exposure cut-off point risk of lung cancer increased significantly. Accordingly, the aim of this study was to review cut-off points above which there was a significant risk of lung cancer due to RCS exposure, or cut-off points below which risk of lung cancer might be very low. In addition, the cut-off points set by various international agencies were also reviewed.

## Materials and methods

### Studies with estimates of Lung cancer risk due to RCS exposure

We performed a systematic review adapted to the PRISMA 2020 (Preferred Reporting Items for Systematic Reviews and Meta-Analyses) guidelines [8].

#### Literature search

A literature search was made in the PubMed database until April 2023, using the following search strategy: “(silica[Title/Abstract] OR crystalline silica[Title/Abstract] OR respirable crystalline silica[Title/Abstract]) AND (lung neoplasms[MeSH Terms] OR lung cancer[Title/Abstract] OR lung tumo*[Title/Abstract])”. We likewise reviewed papers included in the IARC 2012 monograph [1], references cited by papers included, and other systematic reviews on the topic.

#### Inclusion and exclusion criteria

We included published studies that complied with the following PECOS (**P**opulation, **E**xposure, **C**omparator, **O**utcome and **S**tudy design) criteria: (a) studies that were conducted on the adult population (≥ 18 years); (b) who might be exposed to RCS in the workplace; (c) versus those who were not exposed or were exposed to the lowest RCS exposure category; (d) that might have estimated risk of lung cancer mortality or incidence (whether as odds ratios (ORs), relative risks (RRs) or hazard ratios (HRs), accompanied by their 95% confidence intervals (95%CIs)) according to different levels of occupational exposure to RCS. Exposure levels had to be expressed as numerical categories (mg/m^3^ or µg/m^3^) or otherwise indicate to which cut-off points they referred; and lastly, (e) that had a cohort or case-control design.

We only included papers published from 2005 onwards, since a large proportion of the studies published earlier used population data dating from previous decades when exposure to RCS was much higher [9].

We excluded papers that evaluated combined exposures, that assessed exposure in specific populations (i.e., never-smokers), that included fewer than 10 cases of lung cancer, that did not furnish data with a breakdown by different RCS exposure levels, that worked with hypothetical RCS exposure scenarios, and that were in languages other than English, Spanish, Italian, French or Portuguese. Similarly, narrative reviews, editorials, communications to conferences, and op-ed articles were also excluded.

#### Selection of studies and data-extraction

We reviewed the titles and abstracts of papers identified by the literature search, and read the full text of those that were potentially relevant, in order to ascertain their compliance with the inclusion/exclusion criteria. Studies were selected by two researchers working separately, with any doubts being settled by mutual agreement.

A data-extraction sheet was purpose-designed to include basic information about each study (author, year of publication, country, design, sample size, length of follow-up, type of industry), population characteristics (sex and age), RCS exposure levels, and results (RRs, ORs or HRs, and their 95%CIs). Data were extracted by two researchers working separately, with any discrepancies being settled by mutual agreement.

In cases where studies made a series of adjustments, the risks adjusted for most confounding variables were extracted. When the effect measure was not global but was shown with a breakdown by sociodemographic variables, this measure was extracted. Where a study reported results showing cumulative RCS exposure and mean RCS concentrations, both were extracted. Where studies reported results without a lag or with a lag between cause and exposure, the result without a lag was extracted. When different papers extracted data from the same study/cohort, the most recent paper was chosen; however, in cases where they reported different RCS exposure measures, both studies were maintained.

#### Analysis of results

Due to the wide variability in exposure categories between studies, we performed a qualitative synthesis of the studies included.

### RCS exposure limits set by International agencies

We conducted a Google search of the websites for limits set by different international agencies concerned with occupational health. The limits set in the following countries were reviewed: USA, Canada, Japan, Australia, Germany, United Kingdom, The Netherlands, Belgium, Denmark, Finland and Spain.

## Results

### Studies with estimates of Lung cancer risk due to RCS exposure

**Fig. 1:**
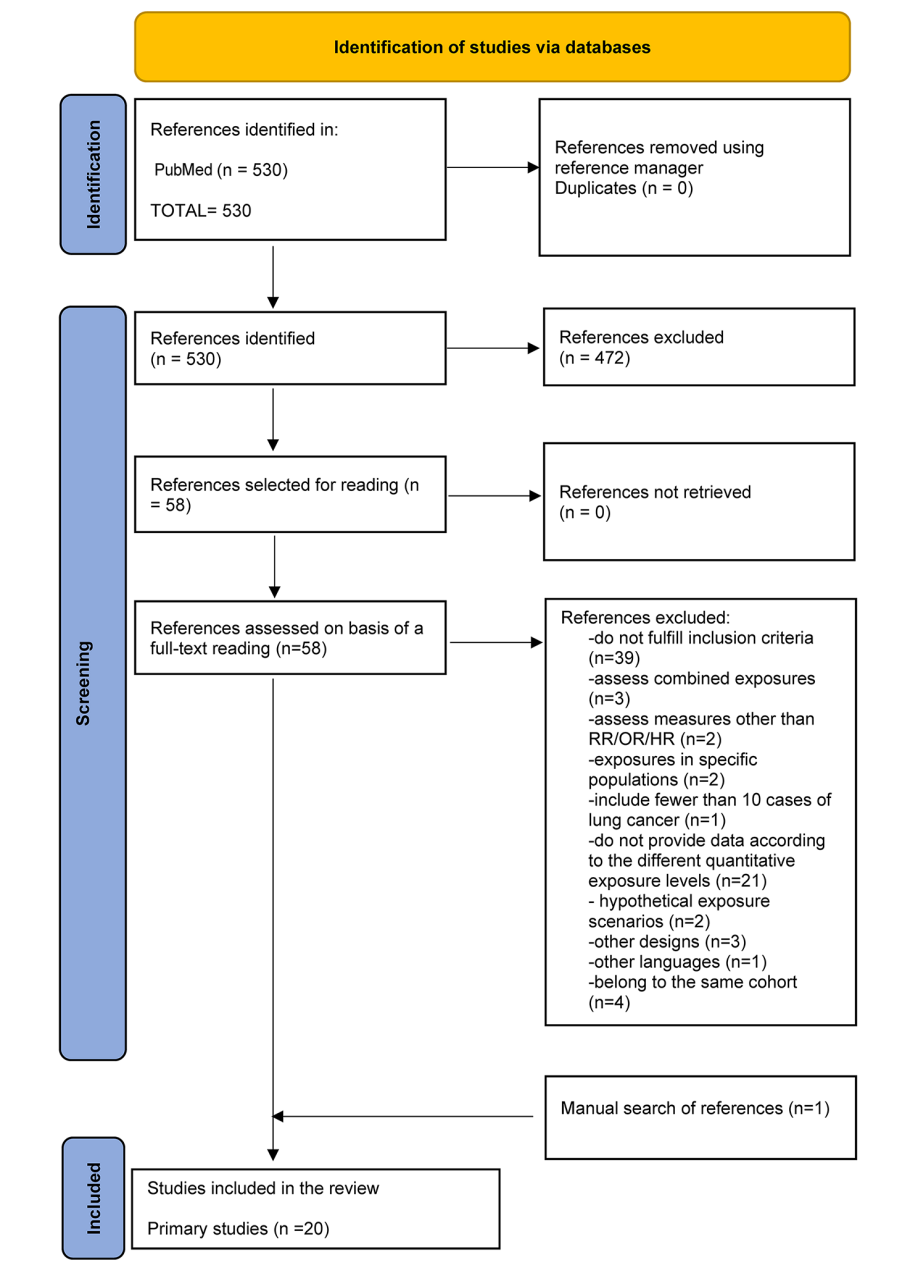
Flowchart of papers included

A total of 530 PubMed entries were obtained. After examination of the titles and abstracts, 58 studies were judged eligible for a full-text review; and of these, 19 were included. During the review of studies to be included, two systematic reviews relating to the study topic were located [6, 7]. The papers covered by both of these reviews were examined, and one study was included. A total of 20 studies were ultimately included. Figure 1 shows the flowchart of the studies included.

#### Characteristics of the studies included and of the study population

Of the 20 studies included, most were cohort studies (n = 14) [10–23] and the rest were case-control studies [9, 24–27], one of which was nested in a cohort study [28]. The studies were carried out in the USA (n = 7), China (n = 2), Europe (n = 10), and one was undertaken in both Europe and Canada. In the cohort studies, the longest follow-up period was 71 years [23] and the shortest, 25 years [12].

The total sample size of the studies was 252,994 subjects. In 10 studies, the participants were men, and the remaining studies included both men and women. In one of the 10 studies that included both sexes, the percentage of women was higher than that of men [11]. A minimum time of RCS exposure was established in 14 studies, i.e., one year in 10 studies, 3 years in two studies [9, 14], and 6 months in a further two studies [11, 17]. Most studies focused on one type of industry, with mining being the most frequent (Tables 1 and 2).

**Table 1:** Description of the main characteristics of the studies included that assess risk of lung cancer mortality related with exposure to respirable crystalline silica

Author	Study area	Design	Sample size	Follow-up time/data-collection	Population characteristics	Minimum time of exposure	Type of industry
Brown et al. 2005	United Kingdom	Cohort	2703	1950–2001	M: 2365 (87.5%)	1 year	Sand
					Mean age of death: 64.6 years		
McDonald et al. 2005	USA	Case-control	Cases: 105	1995–2000	M: 293 (100%)	3 years	Sand
			Controls: 188				
Vacek et al. 2010	USA	Case-control	Cases: 356	1947–2004	M: 1297 (100%)	Not specified	Granite
			Controls: 941				
Mundt et al. 2011	Germany	Cohort	17,573	1938–2005	M: 8183 (47.0%)	6 months	Porcelain
Olsen et al. 2012	USA	Cohort	2650	1945–2004	M: 2474 (93.4%)	1 year	Mining
Sogl et al. 2012	Germany	Cohort	58,677	1946–2003	M: 58,677 (100%)	6 months	Uranium
Cherry et al. 2013	United Kingdom	Cohort	4801	1985–2008	M: 4801 (100%)	1 year	Ceramic
Graber et al. 2014	USA	Cohort	8829	1969–2007	M: 8829 (100%)	Not specified	Mining
					Mean age of registration: 45 years		
Gallagher et al. 2015	USA	Cohort	2343	1942–2011	M: 2343 (100%)	1 year	Diatoms
Allen et al. 2015	USA	Nested case-control	Cases: 1706	1960–2010	M: 4820 (94.8%)	Not specified	Taconite mining
			Controls: 3381				
Lai et al. 2018	China	Cohort	7665	1960–2012	M: 6542 (85.4%)	1 year	Iron mining
					Mean age of entering the cohort: 24.8 years		
Wang et al. 2020	China	Cohort	44,708	1960–2003	M:38,221 (85.5%)	1 year	Ceramic and metal mining
					Mean age of entering the cohort: 26.9 years		
Kleischmidt et al. 2022	USA	Cohort	2650	1945–2015	M: 2473 (93.3%)	1 year	Mining

Abbreviations: M: men, USA: United States of America

**Table 2:** Description of the main characteristics of the studies included that assess lung cancer risk related with exposure to respirable crystalline silica

Author	Study area	Design	Sample size	Follow-up time/data-collection	Population characteristics	Minimum time of exposure	Type of industry
Pukkala et al. 2005	Finland	Cohort	33,664	1971–1995	M: 30,137 (89.5%)	Not specified	Assorted
					Age range: 20–65 years		
Cassidy et al. 2007	Europe	Case-control	Cases: 2852	1998–2002	M: 5956 (75.4%)	1 year	Assorted
			Controls: 3104		Age range: 20–74 years		
Bergdahl et al. 2010	Sweden	Cohort	8320	1958–2000	M: 8320 (100%)	1 year	Iron
Preller et al. 2010	The Netherlands	Case-control	Cases: 1667	1986–1997	M: 3701 (100%)	Not specified	Assorted
			Controls: 2034		Age range: 55–69 years		
Bugge et al. 2012	Norway	Cohort	1166	1953–2008	M: 1166 (100%)	3 years	Carbides
Westberg et al. 2013	Sweden	Cohort	3045	1958–2004	M: 3045 (100%)	1 year	Foundry
Ge et al. 2020	Europe and Canada	Case-control	Cases: 16,901	Not specified	M: 30,056 (79.4%)	Not specified	Assorted
			Controls: 20,965		Age range: 45–80 years		

Abbreviations: M: men, USA: United States of America

A total of 13 studies assessed risk of lung cancer mortality associated with occupational exposure to RCS [9–11, 16–24, 28], and 7 assessed the risk of developing lung cancer [12–15, 25–27]. Most of the studies assessed RCS exposure as cumulative exposure levels in years (n = 14), one assessed it in hours [26], three assessed it as mean concentrations [9, 16, 18], and two assessed it as both cumulative exposure in years and mean concentrations [11, 27]. Tables 1 and 2 show the main characteristics of the studies included.

#### Association between exposure and risk of Lung cancer mortality

In most of the studies, the effect measures calculated were not significant. Five studies obtained significant measurements for some exposure category. In Sogl et al’s study [17], the risk of lung cancer mortality in men began being significant above a cumulative exposure > 10 mg/m^3^-years (RR > 1.47). The study by Gallagher et al. [20] observed a significant HR from 2.6mg/m^3^-years (HR > 1,89) upwards. In Kleinschmidt et al’s study [23], an exposure of 0.224-<0.456 mg/m^3^-years yielded a significant HR (1.85 95%CI 1.09–3.14). The studies by Lai et al. [21] and Wang et al. [22] obtained significant HRs for all categories of exposure. Studies that assessed the risk of lung cancer mortality associated with a mean RCS concentration, reported no significant exposure level (Table 3).

**Table 3:** Risks of lung cancer mortality according to respirable crystalline silica exposure categories set by each study

	**No. deaths**	**Risk**	**95%CI**	**Adjustment**	
*Cohort studies*					
**Brown et al. (2005)**					
* Cumulative exposure (mg/m* ^*3*^ *) years*		*RR*			
<0.13	20	1		Age, period from first employment, employment status, year of starting employment, quarry	
0.13–<0.40	21	1.14	0.60–2.18		
0.40-<1.00	22	1.12	0.58–2.18		
≥ 1.00	19	0.92	0.44–1.92		
**Mundt et al. (2011)**					
* Mean annual concentration (mg/m* ^*3*^ *)*		*HR*			
* Men*				Age, smoking, length of employment	
≤ 0.05	25	1			
> 0.05–0.1	20	2.1	1.1-4.0		
> 0.1–0.15	6	1.3	0.5–3.3		
> 0.15–0.2	12	2.4	1.1–5.2		
> 0.2	11	1.5	0.7–3.3		
* Women*					
≤ 0.05	10	1			
> 0.05–0.1	3	0.5	0.1–1.9		
> 0.1–0.15	5	1.8	0.5–6.3		
> 0.15–0.2	2	1.1	0.2-6.0		
> 0.2	0				
**Mundt et al. (2011)**					
* Cumulative exposure (mg/m* ^*3*^ *) years*		*HR*			
* Men*				Age, smoking	
≤ 0.5	19	1			
> 0.5–1.0	5	0.3	0.1–0.9		
> 1.0–1.5	5	0.4	0.1–1.1		
> 1.5–3.0	16	0.6	0.3–1.2		
> 3.0	29	0.5	0.3-1.0		
* Women*					
≤ 0.5	1	1			
> 0.5–1.0	7	7.8	1.0-63.2		
> 1.0–1.5	3	4.2	0.4–40.4		
> 1.5–3.0	3	2.2	0.2–21.8		
> 3.0	6	3.2	0.4–27.6		
**Olsen et al. (2011)**					
* Mean exposure (mg/m* ^*3*^ *)*		*RR*			
0–<0.015	-	1		Age, race, period since first gainful employment, and mining plant	
0.015–<0.033	-	1.77	0.96–3.29		
0.033	-	1.11	0.54–2.29		
< 0.061	-	1.28	0.58–2.82		
≥ 0.061	-	0.8			
**Sogl et al. (2012)**					
* Cumulative exposure (mg/m* ^*3*^ *) years*		*RR*			
0–0.5	137	1		Radon and arsenic	
0.5–2	283	1.08	0.86–1.31		
2–5	356	1.13	0.89–1.37		
5–10	430	1.05	0.81–1.28		
10–20	936	1.47	1.13–1.81		
20–30	664	2.05	1.51–2.60		
30–56	189	2.79	1.87–3.70		
**Cherry et al. (2013)**					
* Mean concentration (mg/m* ^*3*^ *)*	*Total no.*	*HR*			
	117			Smoking	
< 0.1	-	1			
0.1–<0.15	-	1.07	0.65–1.74		
0.15–<0.2	-	0.76	0.43–1.32		
≥ 0.2	-	0.96	0.58–1.60		
**Graber et al. (2014)**					
* Cumulative exposure (mg/m* ^*3*^ *) years*	*Total no.*	*HR*			
	568			Age, race, year of birth	
< 2.22	-	1			
2.22–3.30	-	1.08	0.85–1.37		
3.31–4.12	-	1.20	0.95–1.52		
≥ 4.13	-	1.17	0.92–1.50		
**Gallagher et al. (2015)**	-				
* Cumulative exposure (mg/m* ^*3*^ *) years*		*HR*			
< 0.4	15	1		Age, calendar year and race	
0.4–<0.9	13	1.38	0.75–2.55		
1.0–<2.6	13	1.02	0.58–1.80		
2.6–<5.6	16	1.89	1.05–3.37		
> 5.6	20	2.03	1.07–3.85		
**Lai et al. (2018)**					
* Cumulative exposure (mg/m* ^*3*^ *) years*	*Total no.*	*HR*			
	262			Sex, year and hiring age, intensity of smoking	
Not exposed	-	1			
≤ 0.4935	-	1.67	1.13–2.47		
0.4935–0.8423	-	1.66	1.19–2.32		
≥ 0.8423	-	1.67	1.22–2.30		
**Wang et al. (2020)**	-				
* Cumulative exposure (mg/m* ^*3*^ *) years*	*Total no.*	*HR*			
	917			Sex, year and hiring age, intensity of smoking	
Not exposed	-	1			
0–1.056	-	1.32	1.07–1.62		
1.057–3.925	-	1.51	1.25–1.83		
> 3.925	-	1.52	1.24–1.87		
**Kleinschmidt et al. (2022)**	-				
* Cumulative exposure (mg/m* ^*3*^ *) years*		*HR*			
< 0.089	29	1		Sex, starting age, race, starting calendar year	
0.089–<0.224	28	1.20	0.70–2.04		
0.224–<0.456	30	1.85	1.09–3.14		
≥ 0.456	29	0.92	0.54–1.58		
*Case-control studies*					
	**Cases**	**Controls**	**Risk**	**95%CI**	**Adjustment**
**McDonald et al. (2005)**					
* Mean concentration (mg/m* ^*3*^ *)*			*OR*		
< 0.07	28	58	1		For matching and smoking
0.07–0.16	30	60	1.01	0.48–2.12	
>0.16–0.26	23	37	1.62	0.75–3.53	
>0.26	24	33	2.36	1.00-5.59	
**Vacek et al. (2010)**					
* Cumulative exposure (mg/m* ^*3*^ *) years*			*OR*		
≤ 0.26	84	241	1		Not adjusted
0.26–0.82	56	176	0.87	0.56–1.29	
0.82–2.09	81	206	1.28	0.90–1.83	
2.09–4.10	74	167	1.29	0.87–1.89	
> 4.10	51	151	0.96	0.60–1.54	
**Allen et al. (2015)**					
* Cumulative exposure (mg/m* ^*3*^ *) years*	*Total no.*		*OR*		
	1706				Taconite, hematite exposure, asbestos and sex
0–0.0372	-		1		
0.0373–0.2063	-		1.04	0.84–1.29	
0.2064–0.5188	-		0.95	0.74–1.22	
≥ 0.5189	-		0.97	0.70–1.35	

Abbreviations: -: not specified, HR: hazard ratio, OR: odds ratio, RR: relative risk, 95% CI: 95% confidence interval

#### Association between exposure and risk of developing lung cancer

In most studies, the effect measures estimated were not significant for a series of exposure categories. Nevertheless, Ge et al’s study [25] reported significance for all categories: hence, the lowest OR was found for > 0-0.39 mg/m^3^-years (OR: 1.15 95%CI 1.04–1.27) and the highest OR for ≥ 2.4 mg/m^3^-years (OR: 1.45 95%CI 1.31–1.60). Other studies reported a significant association for a cumulative exposure ≥ 10mg/m^3^-years (RR: 1.2 95%CI 1.05–1.38) [12], for 2-5mg/m^3^-years (RR: 2.09 95%CI 1.08–4.06) [13], and for a cumulative exposure > 35mg/m^3^-hours (RR > 1.47) [26], in this last case with a lag of 20 years between exposure and development of lung cancer (Table 4).

**Table 4:** Risk of developing lung cancer according to the respective respirable crystalline silica exposure categories set by each study

	**Cases**	**Risk**	**95%CI**	**Adjustment**	
*Cohort studies*					
**Pukkala et al. (2009)**					
* Cumulative exposure (mg/m* ^*3*^ *) years*		*RR*			
Not exposed		1		Age, period, social class, smoking and asbestos	
≤0.9	2999	1.05	0.99–1.10		
1.0–9.9	2339	0.99	0.93–1.05		
≥10	208	1.2	1.05–1.38		
**Bergdahl et al. (2010)**					
* Cumulative exposure (mg/m* ^*3*^ *) years*		*RR*			
Not exposed	14	1		Age and calendar year	
0–2	59	1.62	0.90–2.92		
2–5	27	2.09	1.08–4.06		
>5	12	1.74	0.79–3.85		
**Bugge et al. (2012)**					
* Cumulative exposure (mg/m* ^*3*^ *) years*		*RR*			
*Respirable dust*				Age	
0–3.8	8	1			
3.8–10	18	1.7	0.7-4.0		
10–87	32	2.0	0.9–4.4		
*Respirable quartz*					
0–0.026	10	1			
0.026–0.077	18	1.3	0.6–2.8		
0.077–2.3	30	1.5	0.7–3.1		
*Respirable cristobalite*					
0–0.028	9	1			
0.028–0.093	15	1.2	0.5–2.7		
0.093–2.7	34	2.0	0.9–4.1		
**Westberg et al. (2013)**					
* Cumulative exposure (mg/m* ^*3*^ *) year*	*Total no.*	*HR*			
	53			Age at diagnosis	
<1	-	1			
1–1.9	-	1.01	0.55–1.84		
≥2	-	0.78	0.24–2.57		
	**Cases**	**Controls**	**Risk**	**95%CI**	**Adjustment**
*Case–control studies*					
**Cassidy et al. (2007)**					
* Cumulative exposure (mg/m* ^*3*^ *) hours*			*OR*		
Not exposed 20 years ago	2417		1		Age, sex, center, smoking, educational level, sawdust powder insulation and sawdust
0–9	89	81	1.07	0.77–1.50	
9–35	98	81	1.06	0.75–1.49	
35–200	110	74	1.47	1.04–2.06	
>200	138	74	2.08	1.49–2.90	
**Preller et al. (2010)**					
* Cumulative exposure (mg/m* ^*3*^ *) year*			*RR*		
Not exposed			1		Age, family history of lung cancer, smoking, no. of cig/day, years of smoking, fruit and vegetables and alcohol consumption
>0–<3	148	-	0.95	0.73–1.25	
≥3	62	-	1.47	0.93–2.33	
* Mean concentration (mg/m* ^*3*^ *)*					
Not exposed			1		
>0–<0.075	109	-	0.97	0.70–1.33	
0.075–0.2	75	-	1.21	0.82–1.78	
0.2–0.6	26	-	1.14	0.63–2.05	
**Ge et al. (2020)**					
* Cumulative exposure (mg/m* ^*3*^ *) years*			*OR*		
Not exposed	11,978	16,477	1		Study, age, sex, smoking, list A of occupations
>0–0.39	1113	1128	1.15	1.04–1.27	
0.4–1.09	1221	1120	1.33	1.21–1.47	
1.1–2.39	1231	1122	1.29	1.17–1.42	
≥2.4	1358	1118	1.45	1.31–1.60	

Abbreviations: -: not specified, HR: hazard ratio, OR: odds ratio, RR: relative risk, 95%CI: 95% confidence interval

### RSC exposure limits set by international agencies

Exposure level limits were identified in 11 countries, and in some cases, for a number of agencies [4, 29–43]. Five countries set the RCS exposure limit at 0.05mg/m^3^ for an 8-hour workday (8 h). Whereas some countries, such as the United Kingdom [43] and Belgium, set higher levels that rose to 0.1mg/m^3^ over 8 h [30], others set lower levels, e.g., Japan with 0.3mg/m^3^ [42]. In 2022, Spain’s National Occupational Safety and Health Institute (*Instituto Nacional de Seguridad and Salud en el Trabajo/INSST*) indicated that the daily RCS exposure limit in the work environment should be 0.05mg/m^3^ [35]. In the USA, there were even differences between several of its own agencies: thus, while the Occupational Safety and Health Administration (OSHA) set the exposure limit at 0.05mg/m^3^ for an 8-hour work day [29, 36], the American Conference of Government Industrial Hygiene (ACGIH) set it at 0.025mg/m^3^ for the same period [37] (Supplementary material Table 1).

## Discussion

The studies reviewed show a marked degree of heterogeneity in the exposure categories established, together with widely varying results for lung cancer mortality and incidence due to RCS exposure. Similarly, the studies differ considerably in terms of the occupations assessed and limits analyzed. There are also differences between the limits set by the respective agencies in the different countries; and even in a single country like the USA, differences can be seen in the limits set by its own agencies. Yet despite this absence of consensus, the limit most commonly set by the different authorities is 0.05mg/m^3^.

While several studies observe that high exposure levels have a significant relationship with a higher risk of lung cancer, some studies nevertheless report that the risk in the highest category is lower than that in the lowest categories. This may be due to the fact that the number of workers susceptible to being exposed to high levels is small, thereby accounting for the observed decrease in risk [44]. Although most studies included in this systematic review also reported an increased risk in the lowest exposure categories, these results were not significant in a number of studies. In studies in which mean concentrations were assessed, risk of lung cancer would appear to be low for mean concentrations below 0.07mg/m^3^.

Based on the results obtained in this review, no consensus can be reached on what the RCS exposure limit should be. Studies were however located which discussed the application of different cut-off points. One study stated that setting 0.1mg/m^3^ as the exposure limit would be insufficient but did not indicate a limit that would be considered acceptable [45]. Another study pointed out that setting the limit at 0.1mg/m^3^ would be below the threshold that would trigger pulmonary diseases such as lung cancer [46]. Borm et al. [47] conducted a review in which one of the aspects assessed was the RCS exposure concentration above which there would be a genotoxic effect. They concluded that the lowest dose at which such effects are seen is 40 µg/cm^2^, which is equivalent to 400 µg/m^3^ (i.e., 0.4mg/m^3^).

In relation to the genotoxic effect of the RCS, a review was published in 2011 [48], updating the review conducted by IARC in 1997 [5], in which three mechanisms of carcinogenesis of RCS were proposed. The first was a direct mechanism in which RCS particles interacted directly with DNA causing the release of DNA-damaging free radicals. The second, an indirect mechanism in which RCS depletes antioxidants and increases endogenous oxidative DNA damage or inhibits DNA repair. Third, a secondary mechanism in which RSC produces inflammation and genotoxicity is mediated by, for example, phagocyte derived oxidants. In this review, secondary genotoxicity was proposed as the main mechanism of the RCS for the induction of lung cancer [48]. These mechanisms were consistent with that indicated in the review conducted by IARC in 2012 [1]. However, at that time insufficient data were available to know which mechanism was more likely. In 2018, another review concluded that RCS exposure may induce weak genotoxic effects, generate reactive oxygen species and cause an inflammatory state leading to genotoxicity and organ damage. However, more research is still needed as many of these mechanisms have only been observed in rodents [47].

Three meta-analyses were also published in which the dose-response between exposure to RCS and lung cancer was analyzed. One of these estimated that for every one-unit increase in exposure to silica (mg/m^3^ year), there is an 8% increase in the risk of lung cancer. Even so, the authors indicate that these results must be interpreted with caution because of the heterogeneity among the studies included [49]. In another meta-analysis, the risk of lung cancer was estimated at 22% for a cumulative exposure of 1.0 mg/m^3^-years. Moreover, these authors indicate that there is a risk of lung cancer associated with any level of exposure higher than 1.84mg/m^3^-years [50]. The 2016 meta-analysis [7] observed an increase in risks with the increase in exposure ranges. Hence, for the lowest category of exposure (> 0-≤0.83mg/m^3^-years), risk of lung cancer increased by 19%, and for the highest category (> 8.35mg/m^3^-years) it increased by 36%.

The variability between the studies included is evident. A number of studies indicate a minimum time of working in the industry targeted by the study, and therefore establish a minimum time of exposure to RCS. This minimum time ranges from 6 months to 3 years, though a great proportion of the studies put it at 1 year. Most of the studies are based on data from a cohort with very long follow-up periods, in a population employed in one type of industry. Whereas the shortest follow-up period was 25 years, the longest was 71 years. Yet, in some studies the follow-up period and, by extension, the duration of exposure were not clear, something that amounts to a major limitation. In addition, there was no explanation as to whether measurement of RCS exposure was taken during the workday. Furthermore, the studies targeted different types of industries, with mining being the most frequent. It should be borne in mind here that RCS exposure levels in the different occupations can be highly variable.

A great part of the studies assessed cumulative exposure in years, which is generally calculated on the basis of exposure intensities in the workplace and years worked in that same workplace. It is therefore a measure that takes into account each worker’s intensity of exposure plus his/her duration of exposure. Yet, on being a measure that considers years worked, it should be borne in mind that working conditions may well vary over the course of a work-life. Improvements over time in working conditions, such as ventilation systems, protection equipment which might entail the need to wear high-protection face masks, or routine controls in workers’ exposure to this carcinogen, could serve to influence the value of cumulative exposure.

Another important aspect to bear in mind is age. There are few studies that report on the age of their participants, the ages at which death or the appearance of cases of lung cancer occur, all of which is crucial for attributing a lung cancer risk. In those studies in which the participants’ age range is known, these begin from a younger starting age, like 20 years, or a higher starting age, like 45 or 55 years, and include a population with ages up to 65–80 years. The lack of knowledge in some studies of participants’ ages during measurement of exposure, diagnosis of or death due to lung cancer, is another major limitation.

Another relevant aspect relates to smoking. Some of the studies included report that there may have been an interaction with smoking (observed, for instance, with other occupation-related carcinogens, such as asbestos). Two large-scale case-control studies were undertaken in Canada, which analyzed the effect of exposure to silica and smoking. The first study observed that while the interaction between exposure to silica and smoking was additive, the effect might even be multiplicative [51]. The second study observed that in workers with a 10–40 year history of smoking who were exposed to silica, risk of lung cancer was higher than among smokers who were not exposed. The authors concluded that the interaction between smoking and exposure to crystalline silica was multiplicative [52]. When assessing the effect of RCS exposure on risk of lung cancer, it is therefore important that the confounding role played by tobacco is taken into account. It is likely that the cutting-down or cessation of smoking among workers exposed to RCS may make a significant contribution to decreasing the risk of lung cancer due to such exposure.

Various agencies from different countries have set RCS exposure limits in the workplace. Yet there is no consensus on the cut-off point that should be used to set an RCS exposure limit in the work environment. One study explains that the exposure limit values proposed by different agencies have been set according to risk -rather than health- management criteria [29]. For national or international regulatory agencies, the existing uncertainty appears to focus on a considerably narrow range of exposure, from 0.05 to 0.1mg/m^3^. Some agencies, such as the ACGIH (USA), have arrived at a figure of 0.025mg/m^3^. This also implies that there is consensus among agencies engaged in the prevention of occupational risks, which consists of indicating that from 0.1mg/m^3^ upwards there is a significant risk of disease, and that below 0.025mg/m^3^ this risk would be small.

This study has limitations, linked in the main to the wide variability between studies when it comes to assessing exposure to RCS. This variability is based on important differences in RCS exposures, variability in exposures over time due to improved protective measures, and difference in adjustment variables between studies, apart from the aspects already commented related with age, tobacco exposure and lung cancer diagnosis. Indeed, this wide variability rendered a meta-analysis impossible due to the extremely high heterogeneity. Furthermore, most of the studies assessed the risk of lung cancer mortality, even though it would have been more apposite to analyze the effect on incidence. Our study also has advantages, such as its exhaustiveness in terms of the data collected and analyzed, and its systematic review design, based on the PRISMA guidelines.

In conclusion, there is wide variability between studies in terms of RCS exposure levels. While setting an agreed exposure limit is not possible on the basis of the results obtained, risk of lung cancer appears to be low for mean concentrations of less than 0.07mg/m^3^. There are also differences between the limits set by agencies, though these are within a narrow spectrum ranging from 0.025mg/m^3^ to 0.1mg/m^3^. Most agencies set 0.05mg/m^3^ for an 8-hour workday as their RCS exposure limit. Although there is no precise cut-off point for the existence of risk of lung cancer due to RCS exposure, prevention measures should nonetheless protect workers up to a reasonably feasible limit, which should also take into account cost-effectiveness aspects and detection limits.

### Electronic supplementary material

Below is the link to the electronic supplementary material.

Supplementary Material 1

## Data Availability

The data that support the findings of this study are available from the corresponding author upon reasonable request.

## References

[1] International Agency for Research on Cancer. Arsenic, Metals, Fibres, and Dusts. IARC Monographs: Arsenic, Metals, Fibres, and Dusts in a Review of Human Carcinogens. 2012. 147–168 p.

[2] Martínez González C, Prieto González A, García Alfonso L, Fernández Fernández L, Moreda Bernardo A, Fernández Álvarez R (2019). Silicosis en trabajadores con conglomerados artificiales de cuarzo. Arch Bronconeumol.

[3] Cherry J, Gorman MN, Shafrir A, van Togeren M, Searl A, Sanchez-Jimenez A et al. Health, Socio-Economic and Environmental aspects of possible amendments to the EU Directive on the Protection of Workers from the risks related to exposure to Carcinogens and mutagens at Work. Respirable Crystalline Silica. 2011.

[4] OSHA. OSHA’s Respirable Crystalline Silica Standard for Construction [Internet]. 2017 [cited 2023 Jan 2]. Available from: www.osha.gov.

[5] IARC Working Group on the Evaluation of Carcinogenic Risks to Humans. International Agency for Research on Cancer. Silica, some silicates, Coal Dust and para-aramid fibrils. IARC; 1997. p. 506. PMC5366849 9303953

[6] Shahbazi F, Morsali M, Poorolajal J (2021). The effect of silica exposure on the risk of Lung cancer: a dose-response meta-analysis. Cancer Epidemiol.

[7] Poinen-Rughooputh S, Rughooputh MS, Guo Y, Rong Y, Chen W (2016). Occupational exposure to silica dust and risk of Lung cancer: an updated meta-analysis of epidemiological studies. BMC Public Health.

[8] Page MJ, McKenzie JE, Bossuyt PM, Boutron I, Hoffmann TC, Mulrow CD et al. The PRISMA 2020 statement: an updated guideline for reporting systematic reviews. BMJ. 2021;372. 10.1136/bmj.n71 PMC8005924 33782057

[9] McDonald JC, McDonald AD, Hughes JM, Rando RJ, Weill H (2005). Mortality from lung and Kidney Disease in a cohort of north American industrial sand workers: an update. Ann Occup Hyg.

[10] Brown TP, Rushton L (2005). Mortality in the UK industrial silica sand industry: 2. A retrospective cohort study. Occup Environ Med.

[11] Mundt KA, Birk T, Parsons W, Borsch-Galetke E, Siegmund K, Heavner K (2011). Respirable crystalline silica exposure-response evaluation of silicosis morbidity and Lung cancer mortality in the German porcelain industry cohort. J Occup Environ Med.

[12] Pukkala E, Guo J, Kyyrönen P, Lindbohm ML, Sallmén M, Kauppinen T (2005). National job-exposure matrix in analyses of census-based estimates of occupational cancer risk. Scand J Work Environ Health.

[13] Bergdahl IA, Jonsson H, Eriksson K, Damber L, Järvholm B (2010). Lung cancer and exposure to quartz and diesel exhaust in Swedish iron ore miners with concurrent exposure to radon. Occup Environ Med.

[14] Bugge MD, Kjærheim K, Føreland S, Eduard W, Kjuus H (2012). Lung cancer incidence among Norwegian silicon carbide industry workers: associations with particulate exposure factors. Occup Environ Med.

[15] Westberg H, Andersson L, Bryngelsson IL, Ngo Y, Ohlson CG (2013). Cancer morbidity and quartz exposure in Swedish iron foundries. Int Arch Occup Environ Health.

[16] Olsen GW, Andres KL, Johnson RA, Buehrer BD, Holen BM, Morey SZ (2012). Cohort mortality study of roofing granule mine and mill workers. Part II. Epidemiologic analysis, 1945–2004. J Occup Environ Hyg.

[17] Sogl M, Taeger D, Pallapies D, Brüning T, Dufey F, Schnelzer M (2012). Quantitative relationship between silica exposure and Lung cancer mortality in German uranium miners, 1946–2003. Br J Cancer.

[18] Cherry N, Harris J, McDonald C, Turner S, Taylor TN, Cullinan P (2013). Mortality in a cohort of Staffordshire pottery workers: follow-up to December 2008. Occup Environ Med.

[19] Graber JM, Stayner LT, Cohen RA, Conroy LM, Attfield MD (2014). Respiratory Disease mortality among US coal miners; results after 37 years of follow-up. Occup Environ Med.

[20] Gallagher LG, Park RM, Checkoway H (2015). Extended follow-up of Lung cancer and non-malignant Respiratory Disease mortality among California diatomaceous earth workers. Occup Environ Med.

[21] Lai H, Liu Y, Zhou M, Shi T, Zhou Y, Weng S et al. Combined effect of silica dust exposure and cigarette Smoking on total and cause-specific mortality in iron miners: a cohort study. Environ Health. 2018;17(1). 10.1186/s12940-018-0391-0 PMC5943994 29743082

[22] Wang D, Yang M, Ma J, Zhou M, Wang B, Shi T et al. Association of silica dust exposure with mortality among never smokers: a 44-year cohort study. Int J Hyg Environ Health. 2021;236. 10.1016/j.ijheh.2021.113793 34198202

[23] Kleinschmidt SE, Andres KL, Holen BM, Buehrer BD, Durand G, Taiwo O et al. Mortality among mine and mill workers exposed to respirable crystalline silica. PLoS ONE. 2022;17(10). 10.1371/journal.pone.0274103 PMC9565696 36240241

[24] Vacek PM, Verma DK, Graham WG, Callas PW, Gibbs GW (2011). Mortality in Vermont granite workers and its association with silica exposure. Occup Environ Med.

[25] Ge C, Peters S, Olsson A, Portengen L, Schüz J, Almansa J (2020). Respirable crystalline silica exposure, Smoking, and Lung Cancer Subtype risks. A pooled analysis of case-control studies. Am J Respir Crit Care Med.

[26] Cassidy A, Mannetje A, Van Tongeren M, Field JK, Zaridze D, Szeszenia-Dabrowska N (2007). Occupational exposure to crystalline silica and risk of Lung cancer: a multicenter case-control study in Europe. Epidemiology.

[27] Preller L, Van Den Bosch LMC, Van Den Brandt PA, Kauppinen T, Goldbohm RA (2010). Occupational exposure to silica and Lung cancer risk in the Netherlands. Occup Environ Med.

[28] Allen EM, Alexander BH, MacLehose RF, Nelson HH, Ryan AD, Ramachandran G (2015). Occupational exposures and Lung cancer risk among Minnesota taconite mining workers. Occup Environ Med.

[29] United States Department of Labor. SILICA, CRYSTALLINE, MIXED RESPIRABLE (QUARTZ. CRISTOBALITE, TRIDYMITE) | Occupational Safety and Health Administration [Internet]. 2021 [cited 2023 Jan 2]. Available from: https://www.osha.gov/chemicaldata/278.

[30] FOD. Chemische agentia - Federale Overheidsdienst Werkgelegenheid, Arbeid en Sociaal Overleg [Internet]. [cited 2023 Jan 4]. Available from: https://werk.belgie.be/nl/themas/welzijn-op-het-werk/chemische-kankerverwekkende-mutagene-en-reprotoxische-agentia/chemische.

[31] Health, Authority S. HSA. Our vVision: Healthy, safe and productive lives and enterprises. 2021 [cited 2023 Jan 4]; Available from: https://www.hsa.ie/eng/publications_and_forms/publications/chemical_and_hazardous_substances/2021-code-of-practice-for-the-chemical-agents-and-carcinogens-regulations.pdf.

[32] IFA. IFA - Occupational exposure limit values (OELs).: Binding occupational exposure limit values (OELs) of the European Commission [Internet]. 2022 [cited 2023 Jan 2]. Available from: https://www.dguv.de/ifa/fachinfos/occupational-exposure-limit-values/verbindliche-arbeitsplatzgrenzwerte-der-eu-kommission/index.jsp.

[33] Ministerio de Empleo. Decreto sobre valores límite para sustancias y materiales [Internet]. 2021 [cited 2023 Jan 4]. Available from: https://www.retsinformation.dk/eli/lta/2021/2203.

[34] Health Council of the Netherlands. Health-based Reassessment of Administrative Occupational Exposure Limits [Internet]. 2003 [cited 2023 Jan 2]. Available from: https://www.healthcouncil.nl/documents/advisory-reports/2003/10/22/slate-dust.

[35] INSST. Guía Técnica para la evaluación y prevención de los riesgos relacionados con la exposición a agentes cancerígenos o mutágenos en el trabajo. [cited 2023 Jan 2]; Available from: https://www.insst.es/catalogo-de-publicaciones.

[36] OSHA. Small Entity Compliance Guide for the Respirable Crystalline Silica Standard for General Industry and Maritime. 2017 [cited 2023 Jan 2]; Available from: https://www.osha.gov/sites/default/files/publications/OSHA3911.pdf.

[37] ACGIH, SILICA, CRYSTALLINE — α-QUARTZ AND CRISTOBALITE - ACGIH [Internet]. [cited 2023 Jan 2]. Available from: https://www.acgih.org/silica-crystalline-a-quartz-and-cristobalite/.

[38] IFA. GESTIS International Limit Values [Internet]. 2022 [cited 2023 Jan 4]. Available from: https://limitvalue.ifa.dguv.de/WebForm_ueliste2.aspx.

[39] Government of Ontario. Ministry of Economic Development JC and T. Government of Ontario, Canada. Government of Ontario, Ministry of Economic Development. Job Creation and Trade; 2021.

[40] AIOH. Respirable Crystalline Silica (RCS) Measurement AIOH / NATA Statement of Common Interests Background. [cited 2023 Jan 2]; Available from: https://www.aioh.org.au/resources/publications1/position-technical-papers/technical-papers.

[41] Safe Work Australia. Measuring respirable crystalline silica 2020. [cited 2023 Jan 2]; Available from: www.swa.gov.au.

[42] The Japan Society for Occupational Health. Recommendation of Occupational Exposure Limits (2018–2019): The Japan Society for Occupational Health May, 17, 2018. J Occup Health [Internet]. 2018 Sep 9 [cited 2023 Jan 4];60(5):419. Available from: /pmc/articles/PMC6176032/. 10.1539/joh.ROEL2018 PMC6176032 30259896

[43] Health and Safety Executive. Containing the list of workplace exposure limits for use with the Control of Substances Hazardous to Health Regulations 2002 (as amended). EH40/2005 Work Expo limits [Internet]. 2018 [cited 2023 Jan 2];2002:63. Available from: https://books.hse.gov.uk/.

[44] Chen W, Liu Y, Wang H, Hnizdo E, Sun Y, Su L et al. Long-term exposure to silica dust and risk of total and cause-specific mortality in Chinese workers: a cohort study. PLoS Med. 2012;9(4). 10.1371/journal.pmed.1001206 PMC3328438 22529751

[45] Albin M, Gustavsson P (2020). A silent epidemic: occupational exposure limits are insufficiently protecting individual worker health. Scand J Work Environ Health.

[46] Cox LAT (2011). An exposure-response threshold for lung Diseases and Lung cancer caused by crystalline silica. Risk Anal.

[47] Borm PJA, Fowler P, Kirkland D (2018). An updated review of the genotoxicity of respirable crystalline silica. Part Fibre Toxicol.

[48] Borm PJA, Tran L, Donaldson K (2011). The carcinogenic action of crystalline silica: a review of the evidence supporting secondary inflammation-driven genotoxicity as a principal mechanism. Crit Rev Toxicol.

[49] Lacasse Y, Martin S, Desmeules M, Silicose. silice et cancer du poumon: méta-analyse de la littérature médicale. [cited 2023 Jan 2]; Available from: www.irsst.qc.ca.

[50] Lacasse Y, Martin S, Gagné D, Lakhal L (2009). Dose-response meta-analysis of silica and Lung cancer. Cancer Causes Control.

[51] Vida S, Pintos J, Parent MÉ, Lavoué J, Siemiatycki J (2010). Occupational exposure to silica and Lung cancer: pooled analysis of two case-control studies in Montreal, Canada. Cancer Epidemiol Biomarkers Prev.

[52] Kachuri L, Villeneuve PJ, Parent MÉ, Johnson KC, Harris SA (2014). Occupational exposure to crystalline silica and the risk of Lung cancer in Canadian men. Int J cancer.

