# Supplementary material for: Occupational exposure to respirable crystalline silica and lung cancer: a systematic review of cut-off points
Source: Environ Health. 2023 Nov 30;22:82. doi: 10.1186/s12940-023-01036-0 (PMC10687911; doi:10.1186/s12940-023-01036-0)
Supplement: Supplementary file 1 — Supplementary Material 1 [file 12940_2023_1036_MOESM1_ESM.docx]

**Supplementary material**

**Table 1. Respirable crystalline silica exposure limits set by different International Agencies concerned with occupational health.**

| **Agency** | **Country** | **Limit** |
| --- | --- | --- |
| OSHA, USA | USA | 0.05mg/m^3^ for an 8-hour day |
| NIOSH | USA | 0.05mg/m^3^ for a 10-hour day |
| Cal/OSHA | USA | 0.05mg/m^3^ for an 8-hour day |
| ACGIH | USA | 0.025mg/m^3^ for an 8-hour day |
| AIOH and WHS | Australia | 0.05mg/m^3^ for an 8-hour day |
| OSHA, Canada | Canada | 0.05mg/m^3^ for an 8-hour day |
| JSOH | Japan | 0.03mg/m^3^ |
| European Union | Europe | 0.1mg/m^3^ |
| HSE | United Kingdom | 0.1mg/m^3^ for an 8-hour day |
| IFA | Germany | 0.05mg/m^3^ |
| HCOTN | The Netherlands | 0.075mg/m^3^ for an 8-hour day |
| FOD | Belgium | 0.1mg/m3 for an 8-hour day |
| Ministry of Labor | Denmark | 0.05mg/m^3^ for an 8-hour day |
| Ministry of Social Affairs and Health | Finland | 0.05mg/m^3^ for an 8-hour day |
| INSST | Spain | 0.05mg/m^3^ daily |

OSHA, USA: Occupational Safety and Health Administration; NIOSH: National Institute for Occupational Safety and Health; Cal/OSHA: California Division of Occupational Safety and Health; ACGIH: American Conference of Governmental Industrial Hygienists; AIOH: Australian Institute of Occupational Hygienists; WHS: Safe Work Australia; OSHA de Canada: Occupational Health and Safety Act; JSOH: Japan Society for Occupational Health; HSE: Health and Safety Executive; IFA: Institute for Occupation Safety and Health; HCOTN: Health Council of The Netherlands; FOD: Federal Public Service Employment, Labor and Social Dialogue; *INSST*: *Instituto Nacional de Seguridad and Salud en el Trabajo* (National Occupational Safety and Health Institute).
